# Identification of anti-HBV activities in *Paeonia suffruticosa* Andr. using GRP78 as a drug target on Herbochip^®^

**DOI:** 10.1186/s13020-017-0132-2

**Published:** 2017-04-24

**Authors:** Iao-Fai Lam, Min Huang, Margaret Dah-Tysr Chang, Pei-Wun Yao, Yu-Ting Chou, Sim-Kun Ng, Ying-Lin Tsai, Yu-Chang Lin, Yun-feng Zhang, Xiao-yuan Yang, Yiu-Kay Lai

**Affiliations:** 1 Institute of Biotechnology and Department of Life Science, National Tsing Hua University, Hsinchu, Taiwan; 2 Yunnan Baiyao-Herbcopoeia Laboratory Inc., 51 Xi-Ba Road, Kunming, Yunnan China; 3 Institute of Molecular and Cellular Biology and Department of Medical Science, National Tsing Hua University, Hsinchu, Taiwan; 4 School of Life Science, Yunnan Normal University, Kunming, Yunnan China

**Keywords:** Paeoniaceae, *Paeonia suffruticosa*, Moutan Cortex Radicis, GRP78, Anti-HBV, Herbochip

## Abstract

**Background:**

Herbochip^®^ technology is a high throughput drug screening platform in a reverse screening manner, in which potential chemical leads in herbal extracts are immobilized and drug target proteins can be used as probes for screening process [BMC Complementary and Alternative Medicine (2015) 15:146]. While herbal medicines represent an ideal reservoir for drug screenings, here a molecular chaperone GRP78 is demonstrated to serve as a potential target for antiviral drug discovery.

**Methods:**

We cloned and expressed a truncated but fully functional form of human GRP78 (hGRP78^1-508^) and used it as a probe for anti-HBV drug screening on herbochips. In vitro cytotoxicity and in vitro anti-HBV activity of the herbal extracts were evaluated by MTT and ELISA assays, respectively. Finally, anti-HBV activity was confirmed by in vivo assay using DHBV DNA levels in DHBV-infected ducklings as a model.

**Results:**

Primary screenings using GRP78 on 40 herbochips revealed 11 positives. Four of the positives, namely *Dioscorea bulbifera*, *Lasiosphaera fenzlii*, *Paeonia suffruticosa* and *Polygonum cuspidatum* were subjected to subsequent assays. None of the above extracts was cytotoxic to AML12 cells, but *P. cuspidatum* extract (PCE) was found to be cytotoxic to HepG2 2.2.15 cells. Both PCE and *P. suffruticosa* extract (PSE) suppressed secretion of HBsAg and HBeAg in HepG2 2.2.15 cells. The anti-HBV activity of PSE was further confirmed in vivo.

**Conclusion:**

We have demonstrated that GRP78 is a valid probe for anti-HBV drug screening on herbochips. We have also shown that PSE, while being non-cytotoxic, possesses in vitro and in vivo anti-HBV activities. Taken together, our data suggest that PSE may be a potential anti-HBV agent for therapeutic use.

**Electronic supplementary material:**

The online version of this article (doi:10.1186/s13020-017-0132-2) contains supplementary material, which is available to authorized users.

## Background

Liver infection by hepatitis B virus (HBV) can cause both acute and chronic diseases, which puts human at high risk of death from cirrhosis and liver cancer leading to life-threatening consequences. According to the data of World Health Organization (WHO), hepatitis B remains a major global health problem despite of the availability of highly effective vaccines since 1982 [1]. There are more than 4 million clinical cases of acute HBV infection every year. In low-endemic countries, such as North America and Northern Europe, the estimated prevalence of HBV surface antigen (HBsAg)-positive subjects is less than 2%, while that of high-endemic countries, such as sub-Saharan Africa and China, is greater than 8% [1, 2]. Globally, WHO estimates that approximately 240 million people are chronically infected with hepatitis B (defined as HBsAg-positive for longer than 6 months), and 780 thousand persons die each year for HBV infection—650 thousand from cirrhosis and hepatocellular carcinoma (HCC) due to chronic infection and another 130 thousand from acute hepatitis B [1]. In addition, it is well documented that HCC is one of the most common primary cancers and the third leading cause of death worldwide [3]; and, about 80% of HCC is attributed to HBV and/or HCV infections [4]. In fact, most HCC patients are infected by HBV, but the mechanisms underlying chronic HBV infection and such chronic disease progression remain largely unknown [5]. Early HCC is clinically silent and often well advanced at the first manifestation, only 10–20% of patients are suitable for surgical treatment [6]. Even having undergone surgical treatment, the prognosis of patients with HCC is still poor largely due to the invasion at early stage in HCC [7].

Initial infection of HBV can be prevented by vaccination, while progression of the disease in infected subjects can be prevented by drug treatments. Currently, peginterferon and nucleos(t)ide analogues (NUCs) are common frontline antiviral agents against HBV [2, 8, 9]. However, significant side-effect profile of interferon and drug resistance in NUCs both limits its long-term use and drug resistance in NUCs could limit long-term monotherapy. Moreover, combination therapy with NUCs and peginterferon or two NUCs given for 1 year does not show superiority in durability of response as compared to monotherapy [10]. Therefore, development of novel antiviral drugs and more effective therapies for the treatments of chronic hepatitis B, thus HCC, are urgently needed. In China and Eastern Asian countries including Japan, Korea, Taiwan and Hong Kong, traditional Chinese medicine represents a vast reservoir for anti-HBV drug discoveries [11, 12].

Our previous studies show that ethanolic extract of *Boehmeria nivea* root (BNE) can significantly suppress production of HBV in vitro and in vivo [13–15]. Detailed analysis of the inhibitory mechanism points to the fact that the molecular chaperone 78-kDa glucose-regulated protein (GRP78) is involved. Interestingly, viral core and large surface proteins accompanied with the encapsidated viral DNA were observed to accumulate within the BNE-treated cells. Although GRP78 was suppressed by BNE, stimulation of GRP78 expression by thapsigargin could rescue virus production initially inhibited by BNE. Furthermore, GRP78 siRNA knockdown led to suppression of HBV secretion [14]. Several lines of additional evidence also indicated that GRP78 is involved in the maturation of the large surface protein (HBsAg) and the viral particle of HBV in infected cells [16–19]. Taken together, these observations lead us to conclude that GRP78 may be a target protein for anti-HBV drug discovery and development. However, a competent GRP78 inhibitor or regulator in clinical application is not yet identified. In this study, we aim to identify GRP78 regulators from natural Chinese herbs and potential drug leads for further targeted anti-HBV therapy using the Herbochip^®^ screening platform.

Herbochip^®^, an array-based high throughput screening platform, is an enabling technology for screening active compounds from herbal extracts in the reverse manner. That is, chemical compounds from each herb are firstly fractionated and arrayed onto a surface-activated plastic chip, and hybridization is performed using a biotinylated protein target coupled with streptavidin-cy5 visualization system [20–22]. The platform has been successfully employed to screen herbal fractions that bind to protein targets such as cytochrome P450 3A4 (CYP450 3A4) and tumor necrosis factor-alpha (TNF-α) [21, 22]. These results lead to identification of CYP450 3A4 and TNF-α inhibitory activities in *Sophora flavescens* and *Geranium wilfordii*, respectively, which will then be used in the design of regimens for the treatment of HIV infection and rheumatoid arthritis [21, 22]. In the most recent study, we describe the latest version of Herbochip^®^ screening platform and report successful identification of the binding activity toward TNF-α in 46 out of 82 selected herbal extracts. The anti-TNF-α and anti-inflammatory effects of the *G. wilfordii* extract were, respectively, confirmed by in vitro and in vivo assays [22]. Herein, we present another screening endeavor using GRP78 as a target, hoping to identify antiviral/HBV activities in the medicinal herbs. Accordingly, we have further validated effectiveness of the Herbochip^®^ drug screening platform using GRP78 as a molecular target, and subsequent experiments on ethanolic extract of *Paeonia suffruticosa* (PSE) lead us to conclude that the extract can be used for the treatment of HBV infection.

## Methods

### Preparation of plant extracts

Forty herbs were used for the primary screening (Table 1). Two of the herbs, namely *Camptotheca acuminata* and *Polygonatum kingianum*, were collected in wild and provided to us by Prof. GD Tao of Xishuangbanna Tropical Botanical Garden, Chinese Academy of Sciences (Mengla, Yunnan, China). The plants were identified by Prof. Tao according to The Pharmacopoeia Commission of PR China [23]. The rest of the plant materials, including *P. suffruticosa*, were more common Chinese herbal medicines and were obtained from the Yunnan Baiyao Group Tianzihong Pharmaceutical Co. Ltd. (Kunming, Yunnan, China). Voucher specimens of all plant materials were stored in our laboratory. For large-scale preparation, plant material of *P. suffruticosa* Andr. (*Cortex Moutan Radicis*, the root cortex of *P. suffruticosa*, also known as mu-dan-pi in Chinese herbal medicine) was used. For small-scale preparation, 50 g of plant materials was extracted with 1 l 50% ethanol (1:20 w/v) at room temperature for 3 days. The ethanolic extracts were evaporated to 25 ml under reduced pressure (R200, Buchi, Swiss) at 60 °C and then adjusted to a concentration of 1 g/ml raw drug in 50% ethanol before used. For large amount of *P. suffruticosa* extract (PSE) to be used in subsequent experiments, the starting material was increased to 3 kg and processed according to the same protocol.

**Table 1:** Hybridization reactivityof herbal extracts using GRP78 as a probe on herbochips

Herbal extracts	Parts used	Specimen number	Hybridization reactivity
*Acanthopanax senticosus*	Root and stem	A1208A0350	−
*Agrimonia pilosa*	Whole plant	G0709A0368	+++
*Atractylodes chinensis*	Root and stem	A0604A0084	++
*Bupleurum candollei*	Stem and leaf	H0909B0187	−
*Camptotheca acuminata*	Stem	B1108C0462	−
*Cassia obtusifolia*	Seed	F1004C0160	−
*Cnidium monnieri*	Seed	F1104C0161	−
*Coptis chinensis*	Root	A0804C0142	−
*Cornus officinalis*	Peel	M0303C0139	++
*Cuscuta chinensis*	Seed	F0305C0168	−
*Dioscorea bulbifera*	Tuber	B0204D0052	++
*Eclipta prostrata*	Stem and leaf	H0909E0159	−
*Equisetum hiemale*	Stem	B0507E0160	−
*Ganoderma lucidum*	Sporophore	G0509G0132	−
Herbal extracts	Parts used	Specimen number	Hybridization reactivity
*Hedyotis diffusa*	Stem and leaf	H0109H0148	−
*Isatis indigotica*	Root	A0703I0013	−
*Isatis indigotica*	Leaf	C1003I0015	−
*Lasiosphaera fenzlii*	Sporophore	G0707L0177	++
*Ligustrum lucidum*	Fruit	E0704L0041	−
*Lycium barbarum*	Fruit	E1009L0162	−
*Lycium chinense*	Root cortex	A1004L0044	−
*Onosma paniculatum*	Root cortex	A1204O0013	++
*Paeonia lactiflora*	Root	A1204P0095	++
*Paeonia suffruticosa*	Root cortex	A0305P0100	++
*Phellodendron amurense*	Bark	J0704P0079	−
*Picrorhiza scrophulariiflora*	Root	A0604P0082	−
*Pleione bulbocodioides*	Bulb	B0704P0099	−
*Polygonatum kingianum*	Root	A0308P0239	−
*Polygonum cuspidatum*	Root	A0904P0092	++
*Polyporus umbellatus*	Sclerotium	G1208P0337	−
*Prunus persica*	Seed	F0105P0111	−
*Psoralea corylifolia*	Fruit	E0609P0361	+
*Pueraria thomsonii*	Root	A0904P0080	−
*Rhaponticum uniflorum*	Root	A0903R0025	−
*Rubia cordifolia*	Whole plant	G0309R0113	++
*Scrophularia ningpoensis*	Root	A0904S0074	−
*Scutellaria baicalensis*	Root	A0609S0290	−
*Semiaquilegia adoxoides*	Root	A1003S0067	−
*Sophora tonkinensis*	Root and stem	A0709S0294	−
*Trichosanthes kirilowii*	Root	A0105T0045	−

−, negative; +, weak positive, fluorescence value (FV) < 200; ++, moderate positive, FV between 200 and 1000; +++, strong positive, FV > 1000

### Preparation of herbochips

Herbochips were fabricated as previous described [22]. Briefly, each plant extract obtained above was fractionated by a HPLC system (Model 1100, Agilent Technologies, USA) into 96 fractions, which were then collected into 96-well microplates. The samples were then vacuum-dried (Savant, Thermo Scientific, USA) and stored at 4 °C until used. For arraying, the samples in microplates were dissolved in Optifix I buffer [22], spotted onto surface-activated polystyrene plastic chips alongside with positive controls (Cy5-labeled streptavidin) by an automatic arrayer (Biodot A101, Shuai Ran Precision, Taiwan) according to the format specified (Fig. 1 of Ref. [22]). The slides were then dried, blocked with blocking buffer (0.1 M ethanolamine, 0.1 M sodium tetraborate), washed with TBST (50 mM Tris–HCl, pH 7.5; 0.15 M NaCl, 0.05% Tween 20) and water, then dried again [22]. The resulted slides, with HPLC fractions arrayed, were designated as herbochips and named with the herbs used. For instance, PSE-herbochip is a herbochip that was arrayed with PSE. The herbochips were sealed and stored at 4 °C until used.

**Fig. 1:**
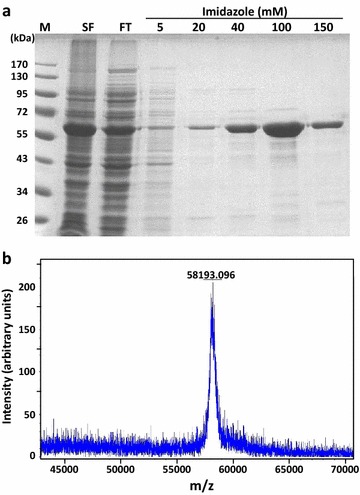
Purification and molecular weight analysis of His-hGRP78^1-508^. **a** *Escherichia coli* culture was induced with 0.5 mM IPTG at 37 °C for 3 h, the supernatant of cell lysate containing His-hGRP78^1-508^ was collected by centrifugation and subjected to nickel-affinity chromatography. His-hGRP78^1-508^ was eluted with different imidazole concentration of 5, 20, 40, 100, and 150 mM. Aliquots of each fraction were analyzed by 12% (w/v) SDS-PAGE. *M* molecular weight marker; *SF* soluble fraction; *FT* Flow-through. **b** Mass determination of His-hGRP78^1-508^ was performed by matrix-assisted laser desorption ionization time-of-flight mass spectrometry (MALDI-TOF MS) operating in the electrospray ionization mode. The data were acquired over the mass-to-charge ratio (m/z) range of 43,000–71,000 under normal scan resolution (*x* axis), the relative intensity (arbitrary units) is shown on the *y* axis. The data from each spectra were summed and deconvoluted

### Preparation of the drug target GRP78 (hGRP78^1-508^)

DNA fragment of hGRP78 was amplified from human lung cDNA library (Stratagene, USA) as described previously [24]. Fragment encoding truncated form of hGRP78^1-508^ was amplified by polymerase chain reaction (PCR) with forward primer XhoI-grp78 (5′-CCG CTC GAG GAG GAG GAC AAG AAG GAG-3′) and reverse primer grp78-BamHI (5′-CGG ATC CCT AGA TTG TGA TCT TAT TTT TGT TCC C-3′). Amplified PCR product was purified, digested with XhoI and BamHI, and then subcloned into XhoI/BamHI cut plasmid pET15b (Novagen, Germany) in frame with a hexahistidine *N*-terminal tag to generate pET15b-grp78^1-508^. The plasmid was transformed into *Escherichia coli* BL21 Gold (DE3) strain. A single colony was selected to grow in 1 l Luria–Bertani (LB) medium (1% tryptone, 0.5% yeast extract, and 0.5% sodium chloride) containing 100 μg/ml ampicillin at 37 °C until OD_600_ reached 0.4–0.6. Protein expression was induced by addition of isopropyl b-d-1-thiogalactopyranoside (IPTG) to a final concentration of 0.5 mM and incubation at 37 °C for 3 h. Cells were harvested by centrifugation and resuspended in 80 ml of equilibrium buffer (20 mM Tris–HCl, 100 mM NaCl, pH 8.0) supplemented with 1 mM protease inhibitor phenylmethylsulfonyl fluoride (PMSF) and disrupted by a three passages through EmulsFlex-C3 high-pressure homogenizer (Avestin, Canada) at 15,000 psi. Cell lysate was centrifuged at 16,000×*g* for 30 min at 4 °C and the resulting supernatant was purified by Ni-Sepharose 6 Fast Flow (GE Healthcare, USA) equilibrated with 50 ml of equilibrium buffer. Subsequently, the column was washed with 200 ml equilibrium buffer supplemented with 5 mM imidazole, followed by 100 ml equilibrium buffer supplemented with 20 mM to 150 mM imidazole. Protein purity was analyzed by 12% (w/v) SDS-PAGE. Finally, the fraction of His-hGRP78^1-508^ eluted with 150 mM imidazole-containing equilibrium buffer was concentrated and buffer-exchanged to coupling buffer (100 mM NaHCO_3_, pH 8.0) using an Amicon Ultra-15 centrifugal filter unit (Millipore, Germany), and protein concentration was determined by bicinchoninic acid (BCA) protein assay reagent kit (Thermo Scientific, USA).

### Biotinylation of GRP78 and screening for its binding activity on herbochips

For biotinylation, 50 μl BNHS solution (2 mg/ml biotinamidohexanoic acid *N*-hydroxysuccinimide ester in DMSO) was added into 1 mg purified His-hGRP78^1-508^ in 1 ml coupling buffer. The biotinylation reaction was carried out for 1 h on ice and then stopped by adding equal volume of glycine (50 mM in coupling buffer). The biotinylated His-hGRP78^1-508^ (designated as b-GRP78) was then dialyzed against coupling buffer to remove unreacted BNHS and excess glycine. For hybridization, each cave on the chips was covered by a cover glass (22 × 22 mm) under which 20 μl b-GRP78 in TBST (50 mM Tris–HCl, pH 7.5; 0.15 M NaCl, 0.05% Tween 20) or TBST alone was added. The slides were then incubated at 37 °C for 1 h. While placed in a slide washing jar, the slides were washed in TBST (4 × 2 min), rinsed in water (4 × 2 min) on a horizontal shaker at 80 rpm at room temperature, and then dried at 37 °C (30 min). The same hybridization procedure was used in the subsequent reaction with Cy5-labeled streptavidin (SA-Cy5) where 20 μl 2.5 μg/ml SA-Cy5 in TBST was used. The dried slides were scanned by a laser scanner (GenePix4100 A, Axon, USA) and the fluorescent intensity of red spots in the image was analyzed at emission wavelength of 635 nm by GenePix 5.0 Software. The fluorescence value (FV) of each hybridization reaction was then recorded.

### Cells and cell culture

HepG2 (ATCC HB-8065) and AML12 (ATCC CRL-2254) cells were purchased from American Type Culture Collection (ATCC). HepG2 2.2.15 cells were kindly provided by Dr. M.-S. Ho (Academia Sinica, Taiwan). The cells were maintained in Dulbecco’s Modified Eagle Medium containing 4.5 g/l d-glucose and l-glutamine (Gibco, Invitrogen, USA) supplemented with 10% fetal bovine serum containing 1.5 g/l sodium bicarbonate, 0.1 mM nonessential amino acids, 1.0 mM sodium pyruvate, and 100 units/ml penicillin G and 100 μg/ml streptomycin. Cells were incubated at 37 °C in a humidified atmosphere containing 5% CO_2_. G418 was added to the medium for maintenance of HepG2 2.2.15 cells at a final concentration of 200 μg/ml. Cells grown in various culture dishes at 80% confluence were used in all experiments.

### Determinations of cytotoxicity and in vitro anti-HBV activity of the herbal extracts

The cytotoxicity effect of herbal extracts on growth of cells was determined by MTT method (Sigma, USA). The cells were seeded in 96-well culture plates at a density of 5 × 10^3^ cells per well and cultured at 37 °C for 24 h. The culture medium was then removed and replaced with fresh medium supplemented with 50 μg/ml herbal extracts, and cultured for 72 h. Negative control of cell growth was carried out by adding the same volume of 50% ethanol in the medium (final concentration of 0.25%). Three hours prior to termination of the culture, 100 μl MTT (0.5 mg/ml in serum free medium) was added to the monolayer of cells. After incubation at 37 °C for 3 h, medium was removed and 100 μl DMSO was added to each well at 37 °C for 20 min to solubilize the formazan. Cell growth was monitored by measuring absorbance at 570 nm (ImageQuant, GE Healthcare, USA). Cell survivals after treatments were presented as % viability = *A*_Treatment_/*A*_Control_ × 100%, where *A*_Treatment_ and *A*_Control_ indicates the absorbance of the treated and the control samples, respectively. In vitro anti-HBV activity was determined by inhibition of HBsAg and HBeAg secretion in HepG2 2.2.15 cells. HepG2 2.2.15 cells were plated at a density of 5 × 10^3^ cells per well on 96-well plate, cultured, and treated exactly as described above. After treatments, the supernatants were collected, centrifuged at 5000 rpm for 10 min to remove cellular debris, and then immediately used for HBsAg or HBeAg detection. The levels of HBsAg and HBeAg in the culture medium were, respectively, measured by SURASE B-96 (TMB) and EASE BN-96 (TMB) ELISA kits (General Biological Corp. Taiwan) according to the manufacturer’s instructions. Absorbance at 450 nm was measured, and the data were calculated as the percentage of antigen levels by the formula: relative levels (%) = *A*_Treatment_/*A*_Control_ × 100%, where *A*_Treatment_ and *A*_Control_ indicates the absorbance of the treated and the untreated control samples, respectively. All samples were examined in triplicates.

### Animal, animal treatments and measurements of in vivo anti-DHBV activity

The duck hepatitis B virus infection model was employed for assessing the in vivo anti-HBV effects of PSE [25, 26]. Thirty 1-day-old DHBV-negative Pekin Aylesbury ducks (*Anas domesticus platyrhyncos*) were obtained from a commerical hatchery and were held in the animal house facilities of the School of Pharmacology, Fudan University (Shanghai, China). All animal handling procedures were approved by Fudan University animal ethics committee (Additional files 2 and 3). The ducklings were maintained under normal daylight and fed with a standard commercial diet with water ad libitum. After intravenously infected with duck hepatitis B virus (DHBV) for 7 days, 30 DHBV-positive duck were selected and separated into five groups (*n* = 6). Three groups were administered orally in diet with 150, 300, and 600 mg/kg/day PSE. Diet mixed with PBS or ADV (10 mg/kg/day) was served, respectively to the other two groups as negative and positive control. In vivo anti-HBV activity was determined by reduction of DHBV DNA (viral load) in serum and liver in the DHBV-challenged ducklings. Serum samples were collected from treated ducks on days 5, 10, and 13 (3 days after cessation of treatment for 10 days, referred to as day P3 hereafter). Fifty microliters of serum sample were used for DHBV DNA measurement by dot blot analysis using DIG High Prime DNA Labeling and Detection Starter Kit I (Roche Life Science, USA). DHBV DNA in liver was measured by Southern blotting. Five hundred milligrams of duck liver tissues were ground in 6 ml of a buffer [10 mM Tris–HCl, pH 7.6, 100 mM EDTA, 0.5% (w/v) SDS], and then digested with protease K at 55 °C overnight, followed by centrifugation at 13,000×*g* for 10 min. The supernatant was treated with RNase A at 37 °C overnight, and then extracted with phenol/chloroform. DNA was precipitated with ethanol, dissolved in 100 μl ddH_2_O, separated on a 1% agarose gel, and analyzed by Southern blotting using DIG High Prime DNA Labeling and Detection Starter Kit I (Roche Life Science, USA).

### Statistical analysis

All data were expressed as mean ± SD. Comparison between groups was carried out by One-way ANOVA. Results with *p* < 0.05 were considered statistically significant.

### Information of experimental design and resources

Details of the experimental design, and statistics, and resources used in this study were included in the Minimum Standards of Reporting Checklist (Additional file 1).

## Results

### Preparation of GRP78 and herbochip screening

Preparation of biotinylated hGRP78^1-508^ (b-GRP78)—Full length hGRP78 was not able to be expressed in *E. coli* due to its toxicity and insolubility, thus a truncated but fully functional hGRP78^1-508^ was used herein [27]. Figure 1a showed that hGRP78^1-508^ with *N*-terminal His-tag expressed in *E. coli* BL21-Gold (DE3) was successfully expressed upon induction with 0.5 mM IPTG. The protein was purified by a nickel-affinity column chromatography with a yield of 30 mg/l of culture medium and purity of 90% (analyzed by Gel-Pro Analyzer software, data not shown). The molecular weight of recombinant His-hGRP78^1-508^ was determined to be 58,193 Da by matrix-assisted laser desorption ionization time-of-flight mass spectrometry (MALDI-TOF MS) (Fig. 1b). The purified hGRP78^1-508^ was buffer-exchanged and concentrated in coupling buffer (100 mM NaHCO_3_, pH 8.0) using an Amicon protein concentrator (30-kDa cut-off) and subjected to further biotinylation. One milligrams of purified His-hGRP78^1-508^ was biotinylated, renamed as b-GRP78 and used as the probe for subsequent screening on herbochips.

**Fig. 2:**
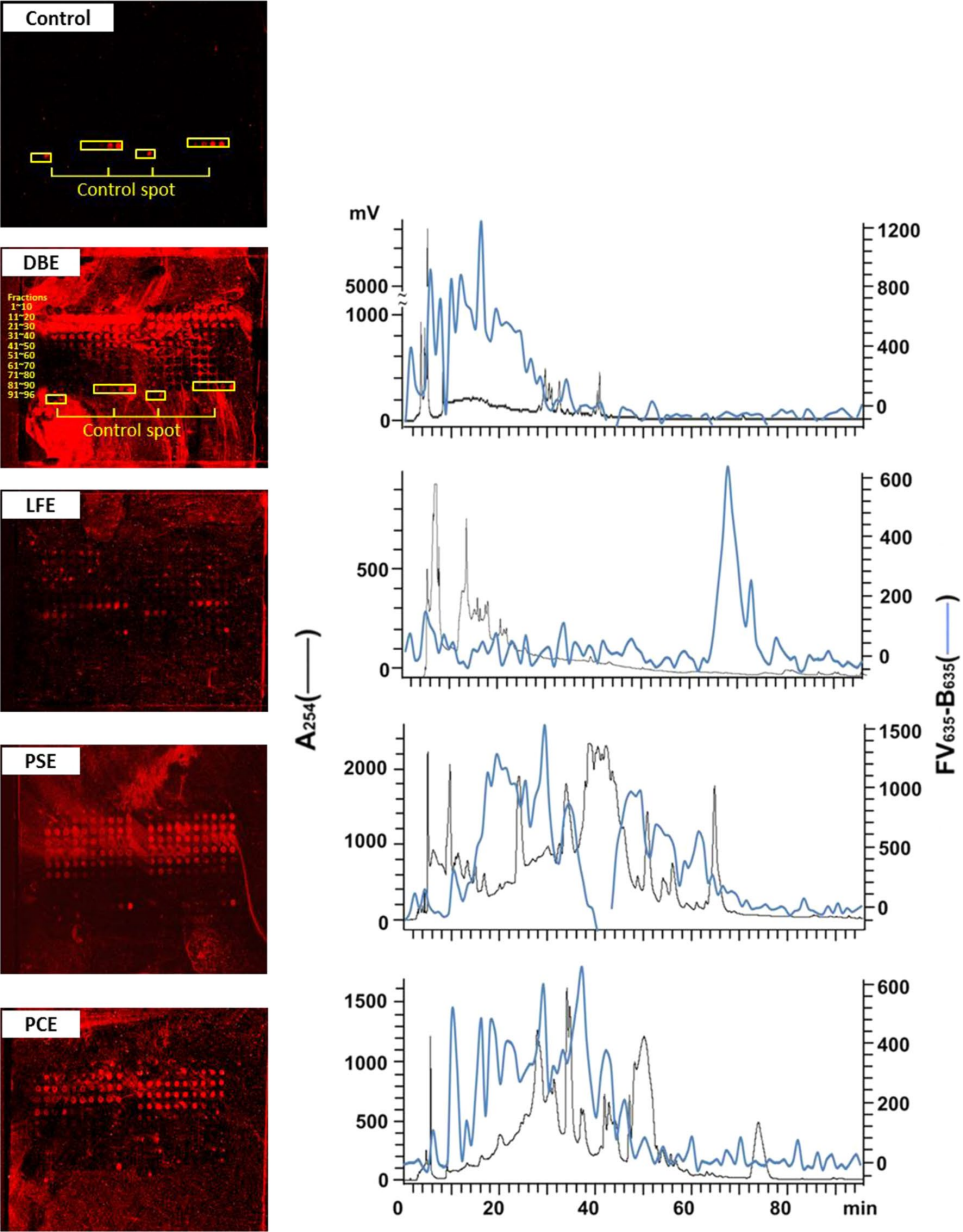
Binding signals of DBE-, LFE-, PSE-, and PCE-herbochips probed by GRP78. *Left panels* the images were visualized by Cy5-labeled streptavidin after the binding of b-GRP78 to DBE-, LFE-, PSE-, and PCE-herbochips, which were fabricated with extracts from *Dioscorea bulbifera*, *Lasiosphaera fenzlii*, *Paeonia suffruticosa*, and *Polygonum cuspidatum*, respectively. The spots in the control were 4, 10, 50, 250 ng/ml biotin in Optifix I, 1 μg/ml SA-Cy5 in Optifix II (positive control) and Optifix I (negative control) as those shown in Fig. 1 of Ref. [22]. *Right panels* the fluorescence intensity of the corresponding images were quantized by a scanner at 635 nm presented (*back line*) together with the original HPLC profile monitored at 254 nm (*blue line*). Positive signals indicated binding activity of the fractions to GRP78

Herbochip screening for GRP78 binding activity—Ethanolic extracts of 40 Chinese herbal medicines (Table 1) were fractionated by HPLC, and 96 fractions of each herb were separately blotted on herbochips, thus a total of 40 herbochips were screened using bGRP78 as the probe. Binding activities were visualized using Cy5-conjugated streptavidin, and the spots with red fluorescent signals indicated target protein binding to the corresponding herbal fractions (Fig. 2, left panels). As mentioned in the Method section, data from four chips containing HPLC fractions from extracts of *Dioscorea bulbifera* (DBE), *Lasiosphaera fenzlii* (LFE), *P. suffruticosa,* and *Polygonum cuspidatum* (PCE) are shown in Fig. 2. The HPLC profiles (*A*_254_) together with quantified binding signals (fluorescence intensity) were also presented (right panels) in which positive binding signals were found in spots corresponding to fractions 6–40 of DBE, 65–73 of LFE, 11–70 of PSE and 11–48 of PCE. These four herbs were then subjected to further studies as mentioned below.

### Cytotoxicity of herbal extracts DBE, LFE, PSE, and PCE on AML12 and HepG2 2.2.15 cells

**Fig. 3:**
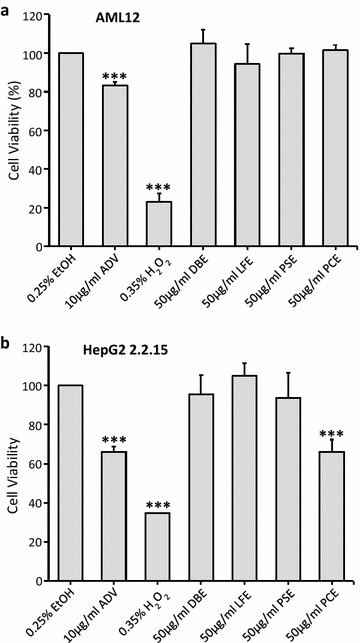
Cytotoxicity of the herbal extracts on AML12 and HepG2 2.2.15 cells. AML12 (**a**) and HepG2 2.2.15 (**b**) cells were treated for 72 h with same dosage (50 μg/ml) of ethanolic extracts of *Dioscorea bulbifera*, *Lasiosphaera fenzlii*, *Paeonia suffruticosa* and *Polygonum cuspidatum* (labeled as DBE, LFE, PSE, and PCE). EtOH and ADV were used as negative and positive controls, respectively. After treatments, cell viability was measured by MTT assay and presented as % of untreated control. The data represent the mean ± SD (*n* > 3). ***p < 0.001. *EtOH* 0.25% ethanol; *ADV* 10 μg/ml adefovir; *H*_*2*_ *O*_*2*_ 0.35%

The effects of the above four herbal extracts on cell viability were evaluated by MTT assay using two different cell lines, AML12 and HepG2 2.2.15. AML12 is derived from alpha mouse liver cell and serves as a normal liver cell control. HepG2 2.2.15 is a widely accepted and well-established cell line for the studies of HBV life cycle and potential HBV inhibitor screening since late 1990s [19]. It is derived from hepatoma HepG2 cells and integrated with a greater than 1-unit-length HBV genome, which not only produces HBV particles but also HBsAg and HBeAg in a continuous manner. Adefovir dipivoxil (ADV), an adenosine monophosphate analog, is approved by US Food and Drug Administration for treatment of hepatitis B. It is an inhibitor of HBV DNA polymerase and competes with natural substrate deoxyadenosine triphosphate; hence, it was used as a control drug. The results showed that there is no suppression of cell viability in AML12 cells treated with extracts from *D. bulbifera* (DBE), *L. fenzlii* (LFE), *P. suffruitcosa* and *P. cuspidatum* (PCE) at a concentration of 50 μg/ml for 3 days, suggesting that these extracts were not cytotoxic to AML12 cells (Fig. 3a). In HepG2 2.2.15 cells, however, treatment with PCE at 50 μg/ml for 3 days resulted in a 33.94% reduction of cell viability; no effect was observed in the other three herbal extracts (Fig. 3b).

### In vitro anti-HBV activity of herbal extracts in HepG2 2.2.15 cells

**Fig. 4:**
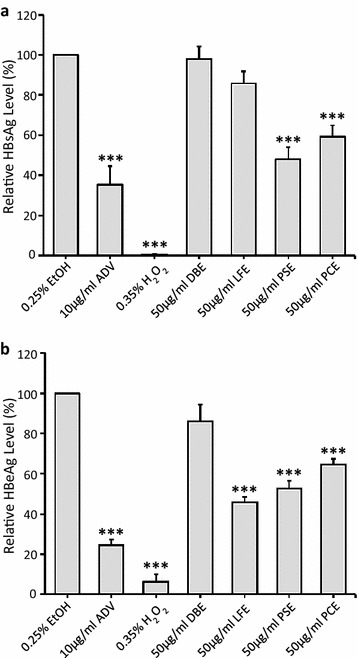
Inhibition of HBsAg and HBeAg secretion by treatment with the herbal extracts on HepG2 2.2.15 cells. HBeAg and HBsAg producing HepG2 2.2.15 cells were treated for 72 h with same dosage (50 μg/ml) of ethanolic extracts of *Dioscorea bulbifera*, *Lasiosphaera fenzlii*, *Paeonia suffruticosa,* and *Polygonum cuspidatum* (labeled as DBE, LFE, PSE, and PCE). EtOH and ADV were used as negative and positive control, respectively. After treatments, levels of HBsAg (**a**) and HBeAg (**b**) in the culture media were determined by ELISA and presented as % of untreated control. The data represent the mean ± SD (*n* > 3). ***p < 0.001. *EtOH* 0.25% ethanol; *ADV* 10 μg/ml adefovir; *H*_*2*_ *O*_*2*_ 0.35%

For anti-HBV activity assay, secretion levels of HBsAg and HBeAg in the supernatant of HepG2 2.2.15 medium were determined by ELISA using, respectively, SURASE B-96 and EASE BN-96 (TMB) ELISA Kits (General Biologicals Corp., Taiwan). Secretion of HBsAg antigen was significantly suppressed in cells treated with PSE and PCE, but not DBE and LFE at a concentration of 50 μg/ml for 3 days (Fig. 4a). The inhibition rates in PSE- and PCE-treated cells were 56.32 and 41.77%, respectively. In terms of HBeAg, the inhibition rates in PSE-, PCE-, and LFE-treated cells were 49.55, 37.00, and 56.44%, respectively, while DBE was ineffective under the same treatment protocol (Fig. 4b). However, the inhibitory effect of PCE to HBV secretion might be due to cytotoxicity to HepG2 2.2.15 cells as reported above. Taken together, PSE was non-toxic to both cell lines but inhibited secretion of both HBV antigens significantly. Hence, it may serve as a potential natural inhibitor from Chinese herbs for targeted anti-HBV therapy and is subjected to the following in vivo studies.

### In vivo anti-HBV activity of PSE in ducks

To further confirm antiviral activity of PSE, serum and liver DHBV DNAs of DHBV-infected ducks were measured after treatment with PSE at various dosages (Table 2). It was observed that DHBV DNA level in the serum of infected ducklings decreased upon treatment with PSE. The inhibition rate was, respectively, 24.14, 41.97, and 35.28% at the dosage of 150, 300, and 600 mg/kg/day after 5 days of treatment (Table 2, T5). At the same dosage after 10 days of treatment, the inhibition rate reduced to 14.46, 39.04, and 19.02% (Table 2, T10). Interestingly, on day P3 (3 days after the cessation of treatment), the inhibition rate was 20.01% at a dosage of 300 mg/kg/day PSE (Table 2, P3), similar to that of ADV at a dosage of 10 mg/kg/day (24.22%). Additionally, livers of infected ducks were obtained on day P3, and the DHBV DNA level was measured by Southern blotting. Densitomertic analysis of the signals showed that the inhibition rates of DHBV DNA upon treatment with 150, 300, and 600 mg/kg/day PSE were, respectively, 13.94, 28.72, and 27.55% (Table 2, the rightmost column). Although the differences were not statistically significant, the reductions of DHBV DNA levels in liver were consistent to those in serum. Taken together, these data suggested that PSE possesses in vivo anti-HBV activity with the highest inhibition rate obtained at 300 mg/kg/day. Moreover, the DHBV DNA level in infected ducks treated with PSE at this dosage rebounded to a less extent after the cessation of treatment as compared to the ADV treated group.

**Table 2:** Inhibitory effects of *Paeonia suffruticosa* extract (PSE) on DHBV DNA level in duckling serum or liver

Agent	Dose (mg/kg/day)	Inhibition rate on day of treatment (T) or post-treatment (P) (%)			
		Serum			Liver
		T5	T10	P3	P3
Adefovir dipivoxil (ADV)	10	83.72***	93.11***	24.22**	46.52***
*Paeonia suffruticosa* extract	150	24.14**	14.46*	0.69	13.94
	300	41.97*	39.04	20.01	28.72
	600	35.28	19.02	2.38	27.55

* *p* < 0.05, ** *p* < 0.01, *** *p* < 0.001

## Discussion

Herbal medicines were used in China and other ancient civilizations for thousands of years; however, most of the mechanisms of their therapeutic effects remain unknown. Herein, we further demonstrated that using the target-based Herbochip^®^ screening platform indeed greatly facilitated discovery and development of herbal drugs for therapeutic uses. As described, the Herbochip^®^ technology reported here is basically a reverse screening method, in which potential chemical leads in herbal extracts have been immobilized and drug target protein is used as the probe for screening process. The success of this technology may be attributed by direct interaction between herbal extracts and diseased-related target proteins, which increase higher possibility of those extracts to act as a regulator to diseases. Prior to the current study, we have examined cytochrome P450 3A5 [21] and TNF-α [22] and the results, respectively, led to the discovery of *S. flavescens* extract and *G. wilfordii* extract for possible application in anti-HIV and anti-inflammatory drug development [21, 22].

Herein, we extend the utilization of the herbochip technology employing GRP78 as a drug target for anti-HBV activity screening. We cloned and purified hGRP78^1-508^ as a drug target to screen against a herbal library of 40 so-called heat removing medicines. Among 40 herbal species screened, 11 were found to bind hGRP78 in which four herbal extracts were subjected to further studies. At the end of the day, the ethanolic extract of the root cortex of *P. suffruticosa* (PSE, also known as *Cortex Moutan Radicis* or mu-dan-pi in Chinese herbal medicine), one of the positive samples, was demonstrated to be non-cytotoxic and able to suppress HBsAg and HBeAg secretion in HepG2 2.2.15 cells. Also, serum and liver DHBV DNA levels in DHBV-infected ducks significantly reduced after oral supplement of PSE. These results clearly revealed that PSE possessed anti-HBV activity both in vitro and in vivo. *Cortex Moutan Radicis* is a well-known Chinese herbal medicine for heat removing and blood cooling [23]. Modern pharmacological studies indicated that this herb possess, among others, anti-inflammatory [28, 29], anti-diabetic [29, 30], and anti-cancer [31, 32] activities. Nevertheless, this study represents the first report on anti-HBV activity of PSE. The anti-HBV activity of PSE is relatively more specific as compared to that of PCE; because of its low general cytoxicity, further development of PSE as anti-HBV agent for therapeutic used is thus warranted. It is worth to note that GRP78, a molecular chaperone, is not only a target for HBV protein maturation and assembly, but also a target/marker for oncogenesis [33–35]. Therefore, all positive signals in herbochip screening using GRP78 as the probe can be re-investigated for their anti-cancer activities. Some of the experiments are already underway and the results will be reported elsewhere.

In summary, we have demonstrated that GRP78, in addition to cytochrome P450 3A5 and TNF-α, can be used as a probe for anti-HBV drug screening on herbochips. We have also shown that one of the positive signals, namely PSE, possesses in vitro and in vivo anti-HBV activities. Our data suggest that PSE may be a potential anti-HBV agent for further therapeutic use.

## Conclusion

We have validated effectiveness of the Herbochip^®^ drug screening platform and demonstrated that GRP78 can be used as a probe for anti-HBV drug screening on the platform. Subsequent experiments on PSE, one of the positive signals, does not exhibit general cytotoxicity. However, PSE possesses in vitro and in vivo anti-HBV activities. These observations lead us to conclude that PSE may be a potential anti-HBV agent for further therapeutic use.

## Additional files

**Additional file 1.** Minimum Standards of Reporting Checklist.

**Additional file 2.** Ethics approval document of Ducklings experiments.

**Additional file 3.** The ARRIVE Guidelines Checklist.

## Abbreviations

GRP78: 78 kDa glucose-regulated protein
HBV: hepatitis B virus
DHBV: duck hepatitis B virus
DBE: *Dioscorea bulbifera* extract
LFE: *Lasiosphaera fenzlii* extract
PSE: *Paeonia suffruticosa* extract
PCE: *Polygonum cuspidatum* extract
HBsAg: HBV surface antigen
HBeAg: HBV envelope antigen
ADV: Adefovir dipivoxil
