# Supplementary material for: Identification of anti-HBV activities in Paeonia suffruticosa Andr. using GRP78 as a drug target on Herbochip®
Source: Chin Med. 2017 Apr 24;12:11. doi: 10.1186/s13020-017-0132-2 (PMC5402670; doi:10.1186/s13020-017-0132-2)
Supplement: Supplementary file 2 — Additional file 2. Ethics approval document of Ducklings experiments. [file 13020_2017_132_MOESM2_ESM.pdf]

复旦大学药学院抗病毒药物研究室

实验动物伦理审批件

伦药批第（2012-05-KBD-ZP-01）号

项目名称	AP0100 抗乙肝病毒动物体内初步药效学试验				
项目类别	技术服务				
项目来源	云南白药企事业合作			经费（万元）	20.0 万
申办单位	复旦大学药学院抗病毒研究室			单位负责人	周珮
研究部门	抗病毒研究室	项目负责人	周珮	职　称	教授
伦理审查意见					
Δ 同意				Δ	
Δ 修改后同意					
Δ 不同意（项目终止或暂停）					

审批意见

同意！

复旦大学药学院抗病毒研究室

（盖章）

主任（签名）周珮

2012 年 05 月 12 日

Ap0100 抗乙肝病毒动物体内初步药效试验及文献资料

项目负责人：周珮

试验单位：复旦大学药学院

试验参加者：周 伟 黄 海 史训龙 盛德鑫

试验日期：2012.6~2012.8

试验资料保存处：复旦大学药学院

联系人：周 珮

联系电话：02154237431

Ap0100、GR0113 抗乙肝病毒初步药效试验

一、试验目的：

观察 Ap0100（牡丹皮提取物）、GR0113（茜草提取物）体内抗乙肝病毒的药效作用。

二、试验材料和检测方法：

1. 毒株：鸭乙肝病毒（DHBV），新鲜分离的阳性麻鸭血清。
2. 动物模型：北京鸭，一日龄，上海青浦金连种禽场提供。
3. 动物试验分组：

   牡丹皮组：600mg/kg 剂量组，300mg/kg 剂量组和 150mg/kg 剂量组

   茜草组：300mg/kg 剂量组，150mg/kg 剂量组和 75mg/kg 剂量组

4、阳性对照药物：阿德福韦酯 10 mg/kg 剂量组

5、受试药物 Ap0100、GR0113 由云南白药中草药芯片有限公司杨晓源高工提供 。

6、血清抗原检测：Dot blot 法

方法：

取 5ul 血清点膜（硝酸纤维素膜），PBST 溶液洗涤（3*10min），30%小牛血清封闭 15min；加入一抗，室温孵育 2 小时，PBST 溶液洗涤（3*10min）；加入二抗，室温孵育 2 小时，PBST 溶液洗涤（3*10min）；加入显色液显色，显色后以蒸馏水漂洗终止反应；Image Master TotalLab Software 测定斑点像素，计算抑制率。

仪器与试剂：

Image Master VDS 凝胶成像仪（Pharmacia Biotech）

硝酸纤维素膜（GE Healthcare）

Biodancer Hybridization 水浴摇床（NBS）

Arium631 纯水仪（Sartorius）

PBST 溶液：

0.01M PBS + 0.05%Tween 20，pH7.2

显色液：

4-氯-α-萘酚	3mg
甲醇	1ml
0.01M PBS，pH7.2	5ml
30%H_2O_2	5ul

7、血清 DHBV- DNA 检测：地高辛试剂盒（Dot-blot）

方法：

取 50ul 血清点样于膜(阳离子尼龙膜)，变性液处理 30min，中和液 1、2 各处理 15min，120℃烤膜 30min，按地高辛试剂盒（DIG High Prime DNA Labeling and Detection Starter Kit I）说明完成 Dot-blot 杂交显色，显色后以蒸馏水漂洗终止反应；Image Master TotalLab Software 测定斑点像素，计算抑制率。

仪器与试剂：

Image Master VDS 凝胶成像仪（Pharmacia Biotech）

阳离子尼龙膜（GE Healthcare）

DHG-9053A 型电热恒温干燥箱（上海精宏实验设备有限公司）

Hybrigene 杂交仪（Techne）

地高辛试剂盒（Roche）

变性液：

NaOH	0.5mol/L
NaCl	1.5mol/L

中和液 1：

Tris-HCl	1mol/L
NaCl	0.6mol/L

中和液 2：

Tris-HCl	0.5mol/L
NaCl	3mol/L

8、 肝脏 RNA 提取和检测：采用 Trizol 试剂盒提取 RNA，用 Real-time 方法检测肝脏 DHBV copy 数。

方法：

取 100mg 肝脏，加入 1mlTrizol 研磨成匀浆，离心取上清，0.2ml 氯仿抽提，取水相加入 0.5ml 异丙醇沉淀，离心取沉淀，1ml 75%乙醇洗涤，200ul DEPC 水溶解沉淀，按 PrimeScript RT reagent Kit With gDNA Eraser 试剂盒完成逆转录，按 PrimeScript RT Master Mix Perfect Real Time 试剂盒检测 DHBV copy 数。

仪器与试剂：

Biofuge Stratos 高速离心机（Heraeus）

RG-300A Rotor-Gene（Rcorbert）

PrimeScript RT reagent Kit With gDNA Eraser 试剂盒（Takara）

PrimeScript RT Master Mix Perfect Real Time 试剂盒（Takara）

DEPC 水：

0.1%DEPC-无菌水

9、肝脏 DHBV- DNA 检测：地高辛试剂盒（Southern-blot）

方法：

取 500mg 肝脏，加入 6ml 裂解液，研磨至匀浆，蛋白酶 K 消化过夜（55℃），10ml 酚/氯仿抽提，4℃离心取上清，加入 12ml 乙醇沉淀，绕出 DNA，1ml 75%乙醇洗涤，离心取沉淀，加入 300ul RNA 酶水溶液溶解，37℃过夜，500ul 酚/氯仿抽提，离心取上清，1ml 无数乙醇沉淀，离心取沉淀， 0.5ml75%乙醇洗涤，DNA 37℃干燥 30min，加入 100ul 无菌水溶解，琼脂糖电泳检测 DNA 浓度。取 20ugDNA 进行 Ecro I 酶切，琼脂糖电泳分离，胶变性 40min，dH_2O 洗涤，中和液中和 2*30min，20*SSC 转膜（阳离子尼龙膜）18 小时，120℃烤膜 30min，按地高辛试剂盒（DIG High Prime DNA Labeling and Detection Starter Kit I）说明完成 Southern-blot 杂交显色，显色后以蒸馏水漂洗终止反应；Image Master TotalLab Software 测定斑点像素，计算抑制率。

仪器与试剂：

Power PAC300 电泳仪（Bio-Rad）

地高辛试剂盒（Roche）

裂解液：

Tris-HCl	10mmol/L

EDTA	0.1mol/L（pH 8.0）
SDS	0.5%

酚/氯仿：

饱和酚：氯仿：异丙醇 = 25：24：1

变性液：

NaOH	0.5mol/L
NaCl	1.5mol/L

中和液：

Tris-HCl	1mol/L
NaCl	0.5mol/L

20*SSC：

柠檬酸三钠	0.3mol/L
NaCl	3mol/L

三、Ap0100、GR0113 体内抗鸭乙肝病毒药效试验

一日龄北京雏鸭取血检测，阴性鸭静脉感染病毒后 7-10 天取血检测，去除阴性鸭。将阳性鸭按三个剂量随机分组，口服给药，一日一次，另设病毒对照组（给生理盐水）和阳性药物对照组阿德福韦酯 10mg/kg。给药后第 5、10 天和停药后 3 天取鸭血分离血清。停药后第 3 天杀剖鸭，采血、取肝，检测血清抗原及血清 DHBV-DNA，检测肝脏 DHBV-DNA，并提取肝脏 RNA 采用 Real-time 方法检测肝脏中 DHBV RNA copy 数。

四、结果

1、对血清抗原的影响

	时间	平均值	抑制率
**茜草 300mg/kg**	0（天）	12599 ±1224.997	
	5（天）	12679.83±1434.351	0%
	10（天）	11761.26 ±1908.933（p=0.387）	6.6%
	13（天）	13513.53 ±1312.192	0%
**茜草 150mg/kg**	0（天）	15603.83±4848.439	
	5（天）	14298.83 ±3111.413（p=0.591）	8.4%

	10（天）	15408.83±3812.097（p=0.940）	0%
	13（天）	15645.69 ±2847.81（p=0.986）	0%
**茜草 75mg/kg**	0（天）	17825.71 ±2863.617	
	5（天）	20009.2±3559.495	0%
	10（天）	20160.51±3510.867	0%
	13（天）	20586.33 ±1539.606	0%
**牡丹皮 600mg/kg**	0（天）	21645.33 ±5933.101	
	5（天）	16434.83±3143.448（p=0.087）	24.1%
	10（天）	18201.41±3824.76（p=0.260）	15.9%
	13（天）	19766.53 ±5346.253（p=0.577）	8.7%
**牡丹皮 300mg/kg**	0（天）	20492.67±3641.49	
	5（天）	18983.5±2597.621（p=0.428）	7.4%
	10（天）	18066.67±2443.612（p=0.205）	11.8%
	13（天）	19787.5 ±2456.746（p=0.702）	3.4%
**牡丹皮 150mg/kg**	0（天）	21491.5 ±2390.34	
	5（天）	22519.75±1159.865	0%
	10（天）	23996.72±1904.858	0%
	13（天）	27341.52 ±2549.329	0%
**阿德福韦酯 10mg/kg**	0（天）	28191.96 ±8559.847	
	5（天）	22955.41±2379.44（p=0.179）	18.5%
	10（天）	28162.39±2758.868	0%
	13（天）	30050.23±2551.754	0%

各组对抗原均未为显示显著抑制，见附图 1

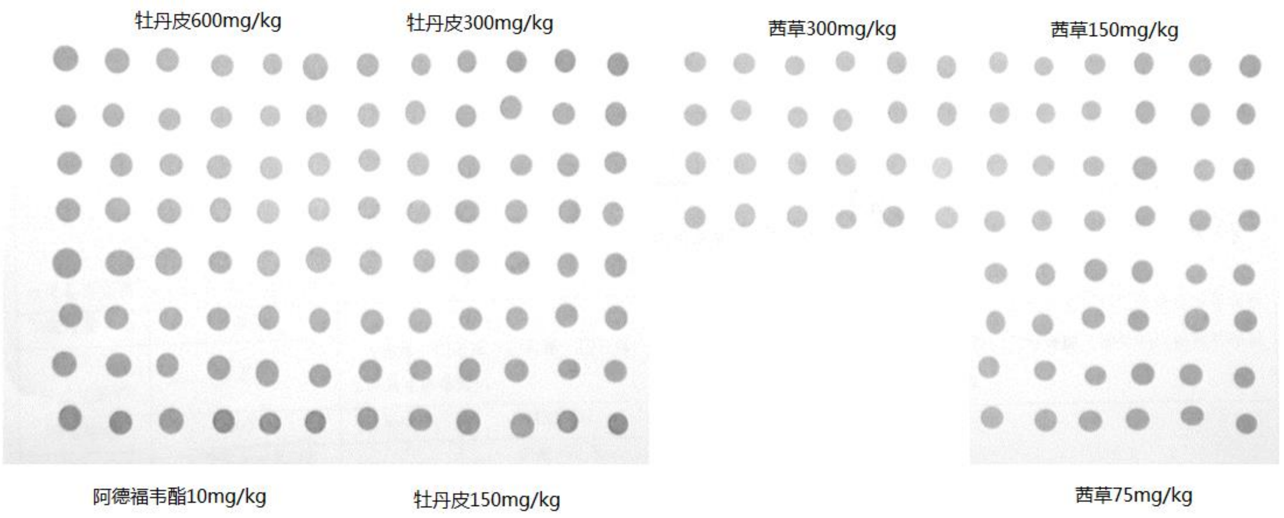

附图 1 对血清抗原的影响

2、对肝脏中 DHBV-RNA 复制的影响

组别	剂量	抑制率
阿德福韦酯	10mg/kg	**98.88%**（p=0.104）
茜草	75mg/kg	**37.46%**（p=0.400）
	150mg/kg	**59.60%**（p=0.275）
	300mg/kg	**89.58%**（p=0.135）
牡丹皮	150mg/kg	**0**
	300mg/kg	**10.09%**（p=0.879）
	600mg/kg	**14.69%**（p=0.848）

以上结果显示：在鸭急性感染模型中，停药 3 天后，茜草提取物对肝脏 DHBV-RNA 水平复制具有明显抑制作用，但与阿德福韦酯 10mg/kg 比较，不如阿德福韦酯。 详见附表 1。

附表 1 对肝脏中 DHBV-RNA 复制的影响

组别		样品号	拷贝数	平均值	SD	抑制率
病毒阳性		24	1.72E+03			
		9	5.34E+02			
		62	1.03E+02			
		64	3.45E+03	1.45E+03	1.50E+03	
阿德福韦酯	10mg/kg	38	4.66E-02			
		89	6.22E+01			
		25	2.59E+00			
		53	1.69E-01	1.62E+01	3.06E+01	**98.88%**
茜草	小剂量	91	1.33E+03			
	75mg/kg	79	7.21E+02			
		78	6.01E+02			
		55	1.25E+03			
		40	8.18E+02			
		17	7.23E+02	9.07E+02	3.06E+02	**37.46%**
	中剂量	6	5.54E+02			
	150mg/kg	30	1.67E+03			
		76	3.62E+02			
		11	3.16E+02			
		84	2.47E+01	5.86E+02	6.36E+02	**59.60%**
	大剂量	59	1.62E+02			
	300mg/kg	22	7.03E+00			
		46	3.61E+02			

		70	7.44E+01	1.51E+02	1.54E+02	**89.58%**
牡丹皮	小剂量	16	3.85E+07			
	150mg/kg	36	2.80E+05			
		7	1.15E+07			
		34	4.56E+07			
		8	4.54E-03	1.92E+07	2.15E+07	**0**
	中剂量	1	4.52E+02			
	300mg/kg	37	4.96E+02			
		74	2.58E+03			
		43	1.70E+03	1.31E+03	1.03E+03	**10.09%**
	大剂量	82	1.01E+03			
	600mg/kg	21	4.73E+02			
		56	7.71E+00			
		61	3.46E+03	1.24E+03	1.54E+03	**14.69%**

3、对血清中 DHBV-DNA 复制的影响

样品	天数	抑制率%
**茜草 300mg/kg**	5	34.64（p=0.007）**
	10	53.77（p<0.001）**
	13	56.01（p<0.001）**
**茜草 150mg/kg**	5	25.43（p=0.021）**
	10	33.36（p=0.003）**
	13	32.79（p=0.002）**
**茜草 75mg/kg**	5	10.77（p=0.136）
	10	11.67（p=0.321）
	13	29.47（p=0.020）**
**牡丹皮 600mg/kg**	5	35.28（p=0.004）**
	10	19.02（p=0.114）
	13	2.38（p=0.800）
**牡丹皮 300mg/kg**	5	41.97（p=0.054）*
	10	39.04（p=0.069）
	13	20.01（p=0.316）
**牡丹皮 150mg/kg**	5	24.14（p=0.009）**
	10	14.46（p=0.055）*
	13	0.69（p=0.964）
**阿德福韦酯 10mg/kg**	5	83.72（p<0.001）**
	10	93.11（p<0.001）**
	13	24.22（p<0.005）**

以上结果显示：在鸭急性感染模型中，停药 3 天后，茜草提取物对血清 DHBV-DNA 水平复

制具有抑制作用，但与阿德福韦酯 10mg/kg 比较，不如阿德福韦酯。但是它对抑制的反跳在停药后也不是很明显。见附图 2。

附图 2 对血清中 DHBV-DNA 复制的影响

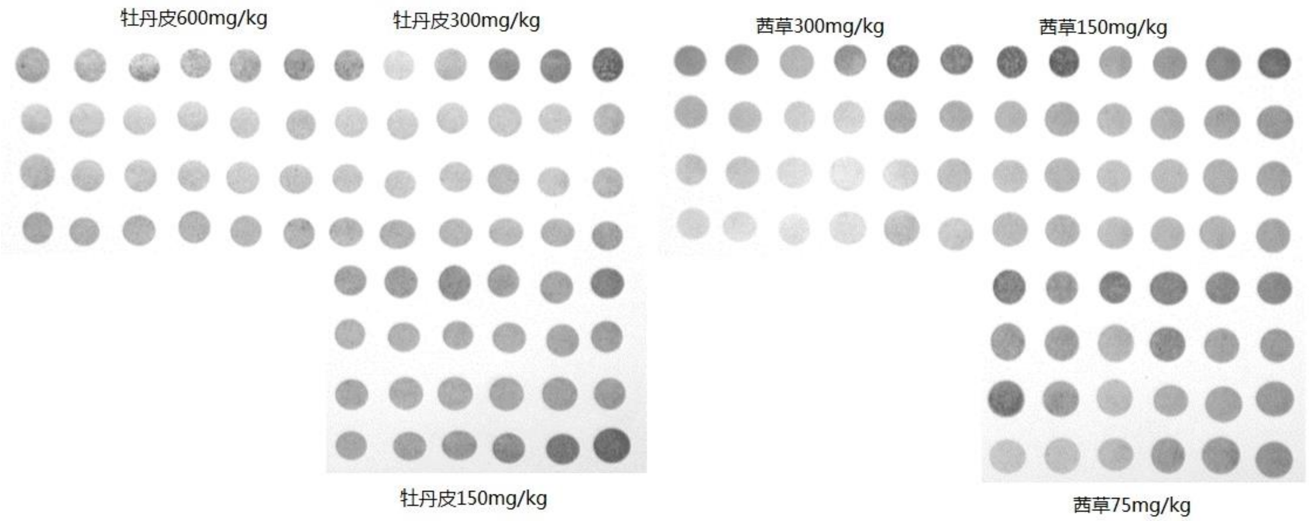

4、对肝脏 DHBV-DNA 复制的影响

组别	剂量	抑制率
**阿德福韦酯**	10mg/kg	**46.52%**（p<0.001）
**茜草**	300mg/kg	**46.06%**（p=0.008）
	150mg/kg	**32.22%**（p=0.002）
	75mg/kg	**10.85%**（p=0.628）
**牡丹皮**	600mg/kg	**27.55%**（p=0.104）
	300mg/kg	**28.72%**（p=0.072）
	150mg/kg	**13.94%**（p=0.479）

茜草和牡丹皮对肝脏 DHBV-DNA 均有抑制复制作用，见如图 3。

附图 3 对肝脏 DHBV-DNA 复制的影响

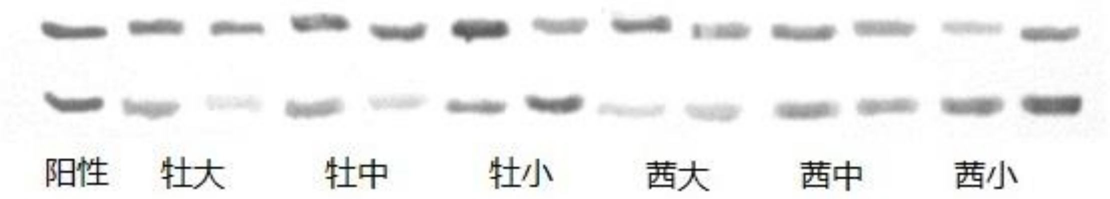

5、讨论：

1、以上结果显示茜草的抑制 DHBV-DNA 作用比牡丹皮略好，但是在抑制 DHBV-RNA 复制中茜草作用更为显著。

2、本次实验仅是初步检测，在蛋白方面的工作还要继续探讨。因为粗提物是不是有影响？

参考文献：

1、 中华人民共和国卫生部药政局．新药临床及临床前研究指导原则汇编．1993。

2、 黄正明，杨新波，曹文斌等．水芹提取物抗鸭乙型肝炎病毒机理的研究．科学通报，1997，17：1863-1867．

3、 赵艳玲，蔡光明，张新金等．赋肝能抗病毒作用实验研究．中国药学杂志，2001，5：305-308．

报告人： 周 珮

报告日期： 2012.8
